# A Case of Pregnancy with Twins, Where the Expulsion of One Child Took Place Seventeen Days before That of the Other

**Published:** 1825-02

**Authors:** F. Bush


**Art. IV.**—***A Case of Pregnancy with Twins, where the expulsion of one Child took place seventeen days before that of the other.*** **Communicated by F. Bush, Esq.**

Mrs. Bryant, of this place, aged forty, the wife of a respectable cheesemonger, being pregnant for the seventh time, experienced, about the latter end of the seventh month of gestation, rigors, pains in the back and loins, accompanied with slight red discharge from the vagina: these symptoms led us to expect premature labour. The pains became more severe on the fourth day (June 2d, 1824), and the expulsion of a fœtus took place; the placenta almost immediately followed, and the discharge was very inconsiderable. The fœtus, which was of the ordinary size of one at six months, was in a state of putrescence, and must have been dead for some days at least.

After the expulsion of this child, the abdomen of the mother was hard, tumid, and, as she stated, the motion of a child in the uterus was distinctly felt; but, as she was totally free from hemorrhage, and almost from pain, I deemed it proper to leave the case to the operation cf nature. Mrs. B. was requested to remain in bed, in a state of as much repose as possible; which she did for some days.

On the 19th of June, she became uneasy, and, after a few hours, labour came on, and a living healthy child was born; and thus the case terminated, without the further occurrence of any thing unusual.

*Frome; November 10th,* 1824.

